# Echocardiographic Diagnosis and Outcome of Pseudoaneurysm of the Mitral-Aortic Intervalvular Fibrosa: Results of a Single-Center Experience in Beijing

**DOI:** 10.1097/MD.0000000000003116

**Published:** 2016-03-18

**Authors:** Jiancheng Han, Yihua He, Xiaoyan Gu, Lin Sun, Ying Zhao, Wenxu Liu, Ye Zhang, Xu Yang, Yan Li

**Affiliations:** From the Department of Ultrasound (JH, YH, XG, LS, YZ, WL, YZ, XY), Beijing Anzhen Hospital, Capital Medical University, Beijing Key Laboratory of Maternal-Fetus Medicine in Fetal Heart Disease, Chaoyang, Beijing, PR China; and Department of General Surgery and The Center for Fetal Research (YL), The Children's Hospital of Philadelphia, Philadelphia, PA.

## Abstract

Pseudoaneurysm of the mitral-aortic intervalvular fibrosa (P-MAIVF) is a rare but potentially fatal entity. Early diagnosis and surgical treatment are particularly important to decrease risk of mortality.

The purpose of this study was to explore the echocardiographic characteristics and outcome of P-MAIVF and to evaluate the potential application of three-dimensional (3D) echocardiography in the evaluation of P-MAIVF.

Clinical and echocardiographic characteristics were retrospectively evaluated in 9 patients with P-MAIVF, 5 of them assessed by 3D echocardiography. P-MAIVF was identified on echocardiography and located in the posterior aspect of the aortic root, expanding in systole and collapsing in diastole. Of the 9 cases examined, 8 were associated with endocarditis and 1 was caused by radio frequency catheter ablation of atrial fibrillation. Five cases were associated with bicuspid aortic valve, and rupture of P-MAIVF was identified in 3 patients. The morphology of P-MAIVF was clearly demonstrated on 3D echocardiography in 5 cases.

In conclusion, echocardiography provides a useful tool in the diagnosis of P-MAIVF. Color Doppler flow imaging can ease identification of the ostium in cases of ruptured pseudoaneurysms. Three-dimensional echocardiography shows the relationship between P-MAIVF and the adjacent anatomic structures.

## INTRODUCTION

Pseudoaneurysm of the mitral-aortic intervalvular fibrosa (P-MAIVF) is a rare but potentially fatal complication that generally occurs secondary to aortic valve endocarditis.^1^ P-MAIVF is located between the mitral and aortic valves and communicates with the left ventricular outflow tract and is prone to rupture, embolize, or even cause coronary artery compression.^2^ Therefore, early diagnosis and surgical treatment is particularly important to decrease risk of mortality.^1,3^

Due to the rarity of P-MAIVF, information on clinical presentation, echocardiographic characteristic, and outcome of diagnosed cases is scarce. The purpose of this study was to retrospectively study the clinical presentation, echocardiographic characteristics, surgical procedures, and outcomes. It was also intended to explore the potential application of 3D echocardiography in the evaluation of P-MAIVF.

## MATERIALS AND METHODS

### Patients

The study population consists of 9 patients with a final diagnosis of P-MAIVF from August 2003 to October 2015. There were 5 male and 4 female patients with age ranging between from 8 to and 54 years (median age of 44 years). This study's protocol was implemented with approval from the review board of Beijing Anzhen Hospital and informed consent was obtained from all patients.

### Echocardiographic Equipment and Study Protocols

Echocardiograms were obtained on all patients with use of the Vivid 7 system (GE-Vingmed Ultrasound AS, Horten, Norway), iE33 ultrasound system (Philips Medical Systems, Bothell, WA) with a 3D matrix-array transesophageal transducer (X7–2t) or the Acuson Sequoia 256 ultrasound machine (Siemens Medical Solutions USA, Inc, Mountain View, CA). All patients were examined by routine 2-dimensional echocardiography. A real-time 3-dimensional transthoracic echocardiography (RT-3D-TTE) examination was performed in 3 patients. Both 2-dimensional transesophageal echocardiography and 3-dimensional transesophageal echocardiography (RT-3D-TEE) examinations were performed in 2 patients for evaluation of involvement of adjacent structures. Color Doppler imaging was used to assess occurrence of perforation and evaluate the atrioventricular or semilunar valves function.

## METHODS OF ANALYSIS

The clinical features, echocardiographic characteristics, surgical procedures, and outcomes were retrospectively analyzed in all patients with P-MAIVF. All echocardiographic images were reviewed and confirmed by 2 experienced readers who were blind to the clinical or surgical data. Analysis of each lesion's characteristics was focused on site, appearance, size, pulsatility, and communication with other cardiac chambers. An intervalvular pseudoaneurysm was defined as an echo-free cavity located posteriorly in the intervalvular fibrosa region just below the aortic annulus, communicating with an adjacent cardiac chamber.^4^ The presence of concomitant valvular vegetations was evaluated and the degree of valvular insufficiency was semi-quantitated using conventional criteria.

## RESULTS

### General Clinical Presentations

Of 9 patients with P-MAIVF, 4 were admitted to the author's hospital due to persistent fever, chest distress, and shortness of breath; 4 cases were transferred to the author's hospital from other healthcare providers due to endocarditis; 1 case was derived from a follow-up visit 1 month after radio frequency catheter ablation of atrial fibrillation. None of these patients had a history of congenital heart disease or connective tissue abnormalities.

### Echocardiographic Characteristics of P-MAIVF

**FIGURE 1:**
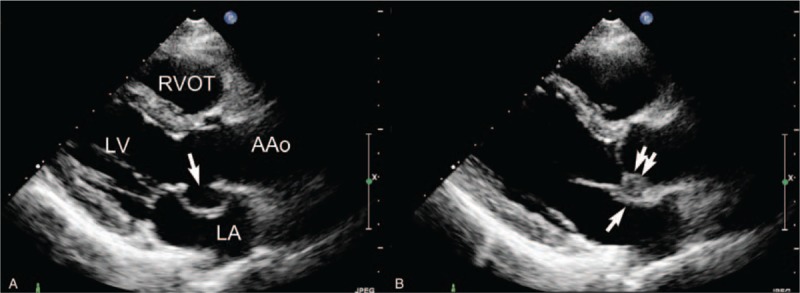
Two-dimensional echocardiography demonstrating P-MAIVF. (A) Left ventricular long-axis view shows a pocket-like echo-free lesion protruding into the left atrium in the mitral-aortic intervalvular fibrosa region during systole. The lesion involved the wall of the noncoronary sinus of Valsalva (single arrow). (B) The lesion collapses during diastole (single arrow); the aortic valve prolapse and vegetation are also observed (double arrow). AAo = ascending aorta; LA = left atrium; LV = left ventricle; P-MAIVF = pseudoaneurysm of the mitral-aortic intervalvular fibrosa; RVOT = right ventricular outflow tract.

**FIGURE 2:**
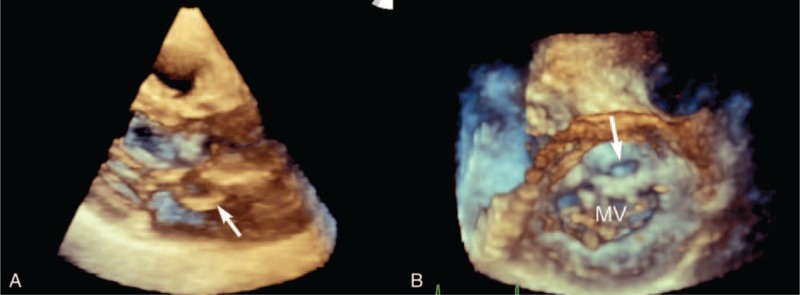
Nonstandard parasternal aortic valve short-axis view showing a pseudoaneurysm in the MAIVF region. (A) Two-dimensional echocardiography shows the expansion of the pseudoaneurysm during systole, protruding into the left and right atrium (star) and the communicating orifice between the lesion and the ascending aorta (arrow) with a diameter of 8 mm. (B) Color Doppler imaging shows the abnormal turbulence signal between the lesion and the ascending aorta (arrow). AAo = ascending aorta; LA = left atrium; MAIVF = mitral-aortic intervalvular fibrosa; P-MAIVF = pseudoaneurysm of the mitral-aortic intervalvular fibrosa; RA = right atrium; RV = right ventricle.

In all patients, a pocket-like lesion was found in the region of the mitral-aortic intervalvular fibrosa, which expanded in systole and collapsed in diastole, in the 2-dimensional left ventricular long-axis view (Figure 1). Color-flow Doppler imaging showed perforations of P-MAIVF wall in 3 patients: in 1 it communicated with the ascending aorta (Figure 2) and in the other 2, with the left atrium. There was no abnormal signal found across the wall of P-MAIVF in 6 patients.

**FIGURE 3:**
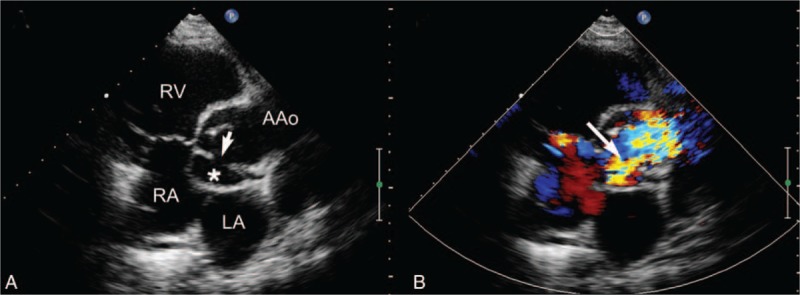
Real-time 3-dimensional transthoracic echocardiography showing P-MAIVF. The pocket-like lesion in the region of mitral aortic intervalvular fibrosa protrudes into the LA in the left ventricle long-axis view. Real-time 3-dimensional transthoracic echocardiography shows the oval orifice between the left ventricular outflow tract and P-MAIVF in the left ventricular view (arrow). MV = mitral valve; P-MAIVF = pseudoaneurysm of the mitral-aortic intervalvular fibrosa.

RT-3D-TTE was performed in 3 patients and demonstrated the relationship between P-MAIVF and adjacent structures (Figure 3A). The oval-shaped communication could be clearly viewed from the left ventricular outflow tract on RT-3D-TTE (Figure 3B).

**FIGURE 4:**
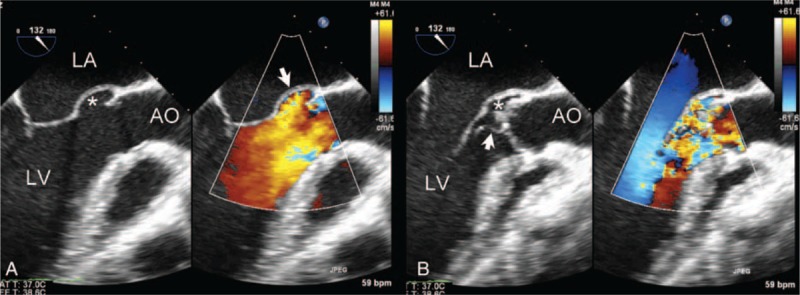
Two-dimensional color Doppler transesophageal echocardiography showing P-MAIVF in the left ventricular outflow tract long-axis view. (A) Two-dimensional TEE showing P-MAIVF expanded (white star) and color Doppler imaging showing a flow filling the cavity with intact wall in systole. (B) P-MAIVF collapsed (white star), aortic vegetation (arrow), and severe aortic regurgitation in diastole. AO = aorta; LA = left atrium; LV = left ventricle; P-MAIVF = pseudoaneurysm of the mitral-aortic intervalvular fibrosa; TEE = three-dimensional transesophageal echocardiography.

**FIGURE 5:**
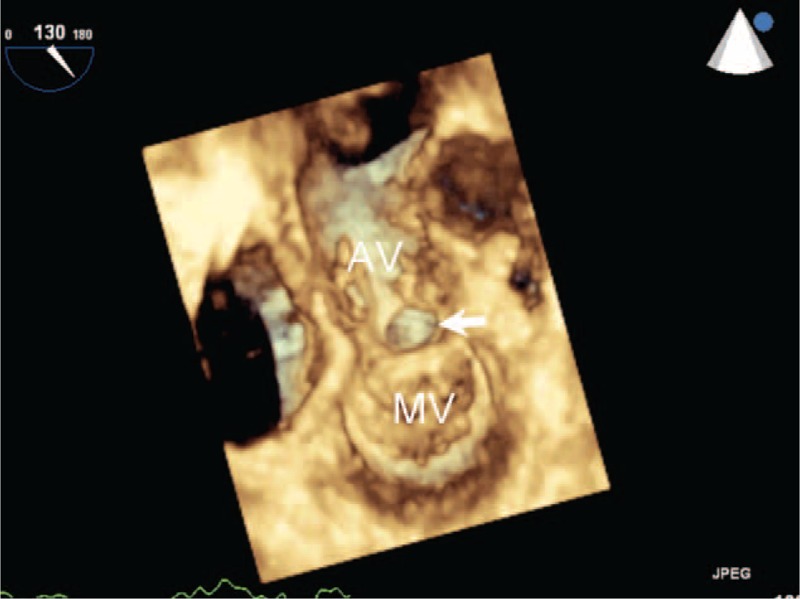
RT-3D-TEE showing the P-MAIVF and aortic vegetation. P-MAIVF with an oval shape (arrow) viewed from left ventricular outflow tract during systole. AV = aortic valve; MV = mitral valve; P-MAIVF = pseudoaneurysm of the mitral-aortic intervalvular fibrosa.

Two-dimensional TEE and RT-3D-TEE were performed in 2 patients to evaluate involvement of adjacent structures. P-MAIVF was very clearly demonstrated and the aortic vegetations were also identified on 2-dimensional TEE (Figure 4). The relationship between P-MAIVF and adjacent structures were also clearly viewed on RT-3D-TEE (Figure 5).

Bicuspid aortic valve anomalies were found in 5 cases (55.6%). Of 8 cases (88.9%) presented with endocarditis, 5 had a combination of aortic valve and mitral valve vegetations and 3 cases only had aortic valve vegetations. Seven cases presented with severe aortic regurgitation, 3 with severe mitral regurgitation, and 2 with severe tricuspid regurgitation.

### Surgical Treatment and Follow-Up

Of all patients, 8 underwent 1 or more of the following surgical procedures: excision or repair of P-MAIVF (7 cases), Bentall procedure—replacement of aortic valve, aortic root and ascending aorta, with reimplantation of the coronary arteries—(2 cases), aortic valve replacement (5 cases), mitral valvuloplasty (2 cases), mitral valve replacement (1 case), tricuspid valvuloplasty (2 cases), sinus repair (1 case) or replacement of the ascending aorta (on case). One patient was not submitted to surgery due to lack of clinical symptoms and P-MAIVF intact wall. Demographic and echocardiographic characteristics and clinical management of patients are summarized in Table 1.

**TABLE 1:**
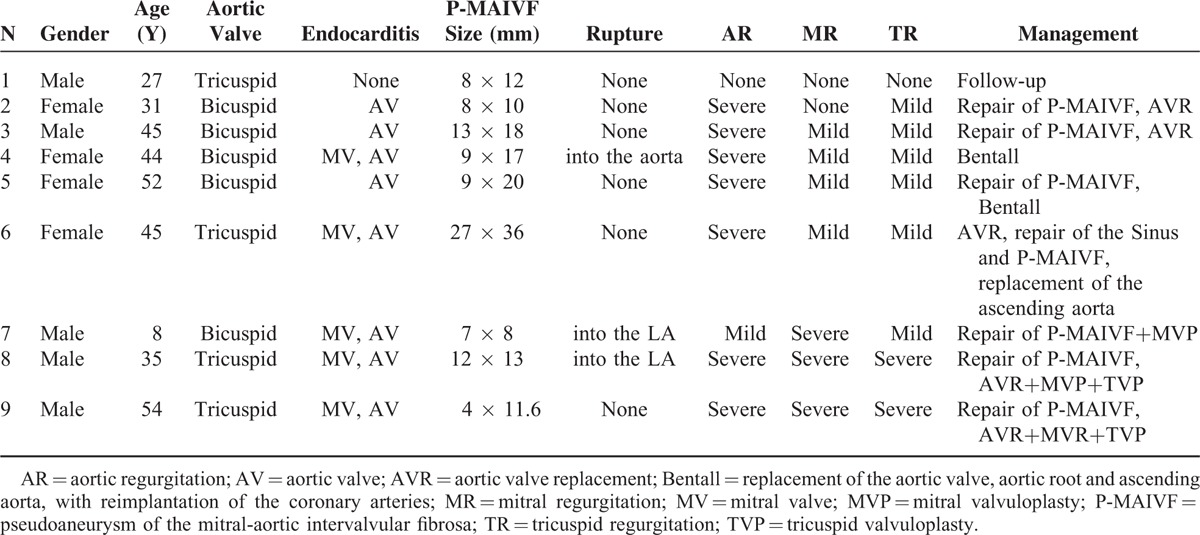
Summary of the Demographic/Echocardiographic Characteristics and Clinical Management of 9 Patients With P-MAIVF

## DISCUSSION

The mitral-aortic intervalvular fibrosa (MAIVF) is a membranous structure with a thin, translucent wall that forms the junction between the left half of the noncoronary cusp, the adjacent third of the left coronary cusp of the aortic valve and the anterior mitral leaflet.^5^ The roof of MAIVF is composed of pericardium and its ventricular side forms the posterior portion of left ventricular outflow tract. MAIVF is composed of a relatively avascular tissue and is prone to infection.

P-MAIVF was first described by Waldhausen.^6^ It commonly occurs in patients with valvular infective endocarditis,^7,8^ especially those with a prosthetic aortic valve.^9,10^ Endocarditis and aortic valve surgery were considered the 2 most frequently associated causative factors for P-MAIVF.^1^ P-MAIVF can also occur in patients without a prosthetic aortic valve,^11^ being associated with chest trauma,^12^ with Takayasu's arteritis^13^ or congenital abnormality.^14^ All patients in this study had their natural valves, which differs from previous reports.^9,10^ The pathogenesis of P-MAIVF still remains largely unknown, but it has been speculated that it may result from either direct extension of endocarditis of the aortic valve or from an “infected” aortic regurgitant jet striking the walls of the LVOT.^15^ Patients with bicuspid aortic valves are more prone to develop this complication, likely because of congenital weakness in the area of the MAIVF.^16^ Of all P-MAIVF patients enrolled in this study, 55.6% (5/9) had bicuspid aortic valves, a higher proportion than that described in a previous study (33%).^1^ In addition, MAIVF infection may also result in abscess formation along the major axis of the aorta, which may subsequently rupture into the LVOT and create a false aneurysm.^17^

P-MAIVF shows a wide spectrum of clinical manifestations, from asymptomatic^18^ to life-threatening cardiac tamponade resulting from pseudoaneurysm rupture.^16^ Affected patients may have *angina pectoris* due to the left coronary artery compression by P-MAIVF,^19^ stroke resulting from embolization of the thrombus^20^ or acute dyspnea from pseudoaneurysm rupture into left atrium.^21^ In the present study, 8 out of 9 cases (88.9%) presented as complications of endocarditis, a higher proportion than that was reported (77%).^1^ The main presentation in this group was symptoms and/or signs of infection from active endocarditis. No cardiac tamponade occurred in these patients.

Echocardiography is a reliable technique to evaluate P-MAIVF. The main echocardiographic characteristic of P-MAIVF is expansion in systole and collapse in diastole. The parasternal left ventricular long-axis view can demonstrate the relationship of the pseudoaneurysm with LVOT or the left atrium. The parasternal aortic short-axis view provides the best view for evaluating the relationship between the lesion and the right atrium or ascending aorta. Color-flow Doppler imaging is helpful to detect ruptures. TEE is more sensitive to detect aortic valve or sub aortic lesions than TTE. In the present study, TEE helped in delineating the pseudoaneurysm and assessing the best surgical repair technique. Therefore, TEE evaluation of structures related to the aortic root is necessary before surgical intervention.^15^ Although suboptimal image quality remains a problem, RE-3D-TTE provides anatomic information that is comparable to that of conventional 2-dimensional echocardiography and provides useful anatomic insight.^22^ In this study, pseudoaneurysm location and the relationship to adjacent structures were clearly shown by real-time 3D echocardiography. RT-3D-TEE can more accurately define the anatomy and morphology of the pseudoaneurysm and may also provide complementary information that is useful for diagnosis and management of P-MAIVF in the operating room.

P-MAIVF should be differentiated from abscess of the aortic ring. However, in practice, it is very difficult to distinguish these 2 lesions. Until imaging technology improves, the phenomenon of expansion in systole and collapse in diastole remains the key point to diagnose P-MAIVF. Compared with P-MAIVF, ring abscesses are smaller, no pulsatile and show no inner flow on color-flow imaging. Whenever necessary, TEE should be performed in order to make a definitive diagnosis.

In recent years, due to improved quality of 3D echocardiography, compared with 2-dimensional echocardiography, it is possible to provide more detailed anatomic information about the lesions.^23^ 3D echocardiography can clearly demonstrate P-MAIVF location as well as size and morphology of the communicating orifice from left ventricular outflow tract view, especially regarding the relationship between the pseudoaneurysm and the mitral or aortic valves.

Surgery should be considered to prevent development of severe complications, even in asymptomatic patients. Surgical procedures include resection and repair of the defect with or without aortic valve replacement or replacement of the aortic root.^24–26^ When the coronary artery is affected, a bypass may be necessary.^27^ Successful percutaneous or transapical closure of P-MAIVF has been reported.^28,29^ Patients with P-MAIVF not submitted to surgery should be monitored.^30^ Although corrective surgery is usually recommended, the appropriate therapeutic approach to this pathology is unclear. Therefore, conservative echocardiographic follow-ups may be a valid and safe alternative to surgery, especially in patients at high risk of post-operative complications.^31^ Once the lesion becomes larger or shows any evidence of perforation, prompt surgical treatment should be considered. During follow-up, no abnormalities were found in the 8 patients that received surgical treatment. One of these patients had a Bentall procedure without excision of P-MAIVF and a closer follow-up was performed. The single patient not submitted to surgery, had no P-MAIVF-related symptoms or evidence of rupture, so follow-up was also recommended.

## LIMITATIONS

The limitations of this study are its retrospective, a single central experience nature and a small number of patients. No echocardiographic parameters about the comparisons between 2-dimensional and 3-dimensional echocardiography were achieved. A large number of patients with systemic analysis are needed.

## CONCLUSIONS

The mitral-aortic intervalvular fibrosa is a relatively avascular structure and offers little resistance to infection. Therefore, when performing an echocardiographic examination in patients with endocarditis, especially in those with bicuspid aortic valve lesions, the sub-aortic structures should be viewed in detail in addition to the aortic valve per se. This study shows that TTE can clearly demonstrate the morphology and dimension of P-MAIVF, color Doppler echocardiography can identify the presence of perforation and 3-dimensional echocardiography can show the morphology of the neck of the lesion as well as its structural relationship with adjacent tissues.
